# Supplementary figures and images for: A higher proportion of ermin-immunopositive oligodendrocytes in areas of remyelination
Source: PLoS One. 2021 Aug 26;16(8):e0256155. doi: 10.1371/journal.pone.0256155 (PMC8389439; doi:10.1371/journal.pone.0256155)

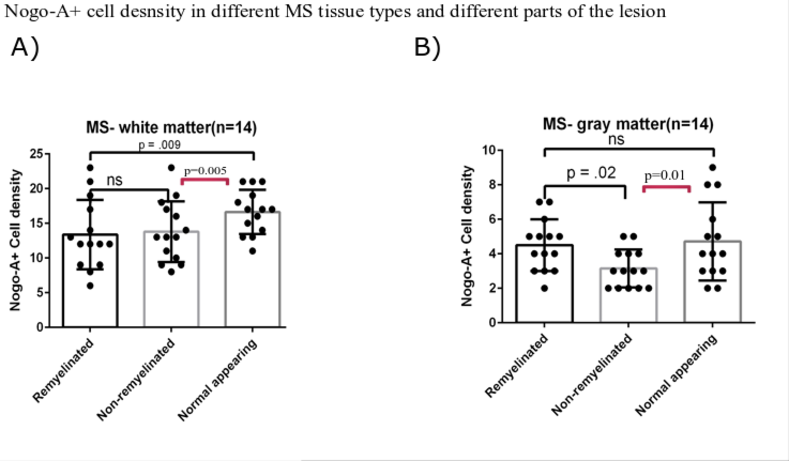

Supplement: S1 Fig — Nogo-A+ cell density in white matter (A) and gray matter (B) in MS-brain. Remyelinated, non-remyelinated and normal-appearing areas are compared. ns: Nonsignificant (p > 0.05). (TIF) [file pone.0256155.s001.tif]
